# Proposing a TAM-SDT-Based Model to Examine the User Acceptance of Massively Multiplayer Online Games

**DOI:** 10.3390/ijerph18073687

**Published:** 2021-04-01

**Authors:** Manuela Linares, M. Dolores Gallego, Salvador Bueno

**Affiliations:** 1 Systems Engineering Program, Universidad de Lima, Lima 15023, Peru; 2 Department of Management and Marketing, Universidad Pablo de Olavide, 41013 Seville, Spain; mdgalper@upo.es (M.D.G.); sbueavi@upo.es (S.B.)

**Keywords:** online games, massively multiplayer online games (MMOG), e-Sports, technology acceptance model (TAM), self-determination theory (SDT), emotions

## Abstract

This study is focused on the massively multiplayer online games’ acceptance. In general, while specialized literature reveals that the online gaming industry has grown strongly in recent years, little evidence is identified on its user acceptance. In this manner, the present study is an attempt to fill this gap. Concretely, two aims are defined: (1) proposing an acceptance model to predict the continuance usage of massively multiplayer online games, and (2) knowing how this continuance usage encourages social well-being. The model proposed employing the structural equation modeling with partial least squares (PLS-SEM) methodology. This PLS-SEM model has been defined using a combination of the technology acceptance model (TAM) and the self-determination theory (SDT), comprising eight constructs: (1) autonomy and competence, (2) relatedness, (3) achievement and challenge, (4) flow experience, (5) perceived enjoyment, (6) social well-being, (7) perceived ease of use, and (8) continuance intention. The findings reveal that continuance intention impacts on social well-being. Moreover, the pivotal role of flow experience for continuance intention has been demonstrated. Additionally, continuance intention has been impacted by the perceived enjoyment, showing that it is a key construct for the acceptance of massively multiplayer online games. Thus, two contributions are highlighted. First, these results provide the gaming industry and software developer companies with considerations on gamers’ motivations in the online game design, in order to stimulate and incentivize its use. Second, the present study can be useful for academicians and practitioners to understand the online gamers’ emotions and well-being, showing some light over their psychology and mental health. Finally, limitations and future directions are exposed.

## 1. Introduction

At current, the online gaming industry generates billions of dollars in revenue. According to researchers [1], gamers of immerse gaming would spend USD 4.5 billion in 2020. In addition, it is expected to reach a 12% of compound annual growth rate (CAGR) for the gaming industry in the 2020–2025 period.

This study is focused on online games. In this respect, considering the used technology to run the game, a traditional classification of video games could be as follow [2]: (1) LAN (Local Area Network) video games are referred to software packages that run on a computer once they have been set up [3]; (2) video web games are those that can be run from an internet browser [3]; (3) online video games or cloud gaming are those that need to be set up on the computer, although a part of the processing is also run on the provider’s server [2,3]; (4) web games charged based on game time [4]; and (5) collaborative games and immersive games, that originally ran through LAN networks. These last have taken great popularity, thanks to the opportunity offered by advances in Information and Communication Technologies (hereafter ICT), allowing high quality streaming. In addition, most of these online games, whether on mobile phones, tablets, laptops, or other devices connected to the internet, are freely distributed.

In recent years, a new type of online games could be added. It is referred to as massively multiplayer online games (MMOG). Most of them are categorized as e-Sports, such as Fortnite, League of Legends, Dota 2, Counter-Strike Global Offensive, Call of Duty, among others [5]. Indeed, MMOG gamers are connected to each other through the internet. These games have become tremendously popular with the Battle Royale competitions, as seen in 2019 with Fortnite, when a teenager won US$ 3 million in the single mode competition [6]. The case of Fortnite is quite remarkable. This game, developed by Epic Games, is extensively widespread, which has more than 250 million registered users [7]. Furthermore, according to a study [8], the MMOG market will reach over US$ 55.5 billion by 2027, with a compounded growth of 6% per year.

In general, numerous studies have been identified in the field of ICT acceptance and motivations. However, while a wide range of investigations focused on different topics related to online games are found, such as quality of online game suppliers [3,9], family behavior [10], gamers characteristics and requirements [11,12,13], purchase intention [14] or social interaction, and subjective norm [4,15,16,17], little research exists on MMOG acceptance. In this report, studies that integrate motivational, emotional, and psychological dimensions have not been detected.

Adopting a user perspective, psychological states, such as flow experience and social well-being [18], could explain the growing tendency to consume these types of online games, and, therefore, their acceptance. In this manner, flow experience is related to the loss of self-awareness, making it impossible for gamers to recognize, for example, changes in their environment [4,19]. Thus, flow experience is based on intrinsic motivations, as well as cognitive absorption, which are more powerful than extrinsic motivations [20]. In this way, it is relevant to evaluate them to reach a better understanding of gamers’ motivations.

### Research Problem and Objectives

Considering the absence of this type of study, some additional research questions are necessary to increase the knowledge about gamers’ motivations in continuance usage of online games, as well as the magnitude of the impact of flow experience and entertainment on their emotions and well-being. Thereby, the aim of this study is twofold:

1. proposing an acceptance model to predict the continuance usage of online games.
2. knowing how this continuance usage encourages social well-being.

In fact, this work proposes to develop an acceptance model for online games, based on the original technology acceptance model (TAM), its extension, known as e-TAM (emotional TAM), and the self-determination theory (SDT). The selection of these theoretical frameworks has allowed to include in the study the variable flow experience [4,19], previously mentioned, among other variables linked with the perceived ease of use, perceived usefulness and perceived enjoyment [10,15,21]. This model was defined using the structural equation modeling with partial least squares (PLS-SEM) method, which allows to evaluate the appropriateness of the constructs incorporated to the model.

The rest of this article is divided into four sections. Section 2 exposes the theoretical frameworks and the hypotheses based on the mentioned theoretical approaches; Section 3 provides the method; Section 4 and Section 5 show the findings and discussions, respectively. In the end, Section 6 exposes the conclusions, contributions, and limitations.

## 2. Research Frameworks and Hypothesis Development

### 2.1. Theoretical Background

In recent years, the ICT development has been accelerated, changing routines for companies and people and ICT-based games are not an exception. Under this premise, some models and theories have been proposed to examine the ICT acceptance. In particular, a range of investigations have developed models to analyze the factors that motivate gamers to continue playing online games. Thus, for example, researchers [11] analyzed the gamers´ attitude and intention to play online games with a model that incorporated variables such as, visual appeal, escape from reality and self-efficiency. Moreover, a study [22], using a seven-factor model (enjoyment, fantasy, escapism, social interaction, social presence, achievement, and self-presentation), determined the influences on continuance intention to play online games. However, despite the existence of these studies, positive effects of the online games use have been poorly studied.

In this respect, focusing on MMOG literature, some studies are identified in the emotional and psychological fields. On the one hand, they frequently analyze the negative effects on the individual’s psychology, mainly addictions. Thus, researchers [23] identified five levels of addictions in MMOG contexts, demonstrating that some MMOG motivations can induce severe addictions. In this particular aspect, researchers [24] observed that addicted MMOG gamers are prone to feeling closer to their avatar rather than their real self. Besides that, regular online interactions with strangers are associated with the negative perception of detraction of time for social interactions and activities with offline friends [25]. Moreover, a study [26] found out that regular MMOG players possess difficulties in the process of identity formation. In addition, MMOG have been analyzed as a source of social anxiety, aggressiveness [27], and psychological dependency [28].

On the other hand, playing games is considered a mode to satisfy the desire of self-affirmation in a group [29]. In this respect, some investigations have analyzed the connections between MMOG players and virtual communities. Moreover, researchers [30] identified that members of MMOG groups consider its connections as transcendent. In this manner, authors [31] observed that MMOG has a positive impact on health because of a higher online social support. Indeed, MMOG is seen as an opportunity for assimilating certain social abilities [32]. In this line, researchers [33] revealed that MMOG players can reach social values playing them.

In this respect, a study [17] evaluated the social interactions between several teams of gamers, affirming its positive impact on gamers´ well-being, although they omitted the continuance intention dimension. Moreover, researchers [13] studied the continuance intention adopting a satisfaction point of view, but they did not incorporate variables, such as flow experience and social well-being. In this line, researchers [34] investigated the impact of the dimensions, escapism, fantasy, role projection, enjoyment, emotional involvement, arousal, and sensory experience on children’s well-being when they play games on smartphone, but without considering variables related to the attitude towards use.

Thereby, all these studies show that there is a lack of an integrative approach that analyzes the MMOG acceptance from three points of view: (1) motivations, (2) emotions, and (3) psychological states, and this study is an attempt to fill this gap. Concretely, a combination of TAM and SDT has been proposed to define the research model. In this respect, the TAM, defined by Davis [35], has been used mostly as a theoretical framework to understand the ICT user acceptance. This framework is frequently applied to define models to foretell and to assess the continuance usage of a wide range of ICT in many fields.

According to a study [35], the TAM includes five dimensions: (1) perceived usefulness, (2) perceived ease of use, (3) attitude toward using, (4) continuance intention, and (5) actual use. The relationships between these constructs are well stablished. Considering these constructs, the level of acceptance towards an ICT depends on user perceptions. In this manner, while perceived usefulness is associated with the individual beliefs about the capacity of an ICT to improve the job performance, perceived ease of use is related to the personal beliefs about the free of effort of an ICT [36]. According to the original TAM proposal, perceived ease of use impacts positively on perceived usefulness and continuance intention, and perceived usefulness impacts positively on continuance intention [35]. In particular, the present study has applied the emotional TAM (e-TAM). This variant of TAM was proposed by researchers [37] in 2019, and it incorporates motivational variables related to achievement, challenge, and well-being, in as much as, psychological motivations and perceptions. E-TAM starts to be applied in research. This is the case of the study conducted by researchers [38] which explores the acceptance factors of social media and the impact on their use for managing disaster proposes. Based on this framework, the present study has replaced the construct perceived usefulness by perceived enjoyment to adapt the model to an entertainment-related context remaining the hypothesized relationships with this fitted variable in TAM. In this manner, the construct perceived usefulness is based on extrinsic motivations, and, therefore, it cannot measure properly the innate intrinsic motivations associated with the feelings of pleasure or entertainment in online games [4,39]. In a similar way, the construct flow experience has been added. It influences positively the attitude and purchase intention of online games [40], showing that the flow experience construct increases the continuance intention to use them [4].

In addition, SDT is a theoretical framework developed by researchers [41] to improve the understanding of the dimensions that incentivize the self-motivation, establishing that the well-being enhanced when individuals do interesting or enjoyable activities [42,43]. Thus, this theoretical framework is suitable for contexts linked to entertainment. In this manner, three constructs could be incorporated to the present study from SDT: (1) need for autonomy, (2) need for competence, and (3) need for relatedness [21]. First, autonomy refers to the self-regulation or self-control of behavior [44,45]. Therefore, this dimension recognizes the capacity of encouraging the development of self-inflicted individual actions. Otherwise, competence is associated with the purpose to become self-sufficient [46]. In this way, an individual with this dimension considers that he or she can carry out tasks effectively [41]. Finally, relatedness is referred to the desire to create social connections [47]. In virtual contexts, relatedness would be associated with the need of collaborating to reach common aims.

**Figure 1:**
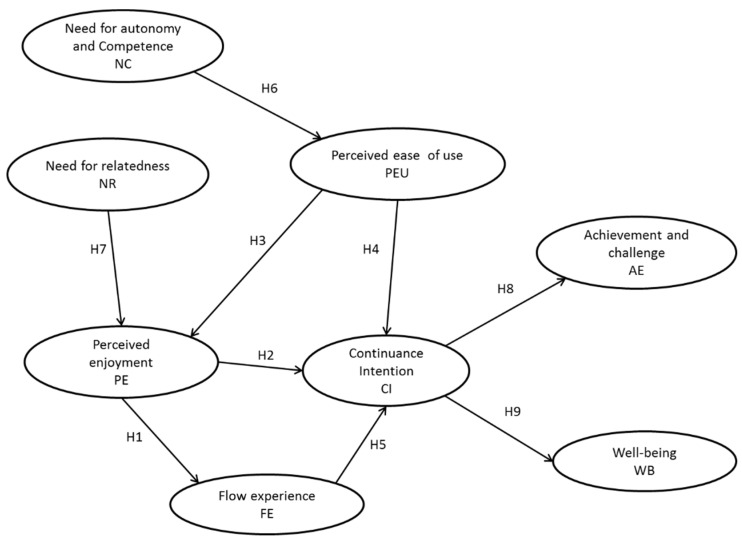
Proposed model. Sources: [19,21,37].

Then, the way of incorporating the constructs come from both theoretical frameworks is shown in Figure 1. In this particular, SDT supported the detection of the external dimensions to e-TAM that determine the proposed acceptance model. In this respect, four variables have been included from the e-TAM model [37]: (1) achievement emotions, (2) challenge emotions, (3) well-being, and (4) perceived value. In this manner, the hypotheses’ development is shown in the next sections.

### 2.2. Hypotheses’ Development

#### 2.2.1. Perceived Enjoyment

Perceived enjoyment is directly related to intrinsic motivations [14]. It tries to measure the satisfaction and pleasure of using ICT [21]. In online games’ contexts, it is demonstrated that users reach satisfactions while they are playing, mainly enjoyment [19]. Moreover, perceived enjoyment shows satisfaction while a user is playing, which would be related to entertainment [39]. Consequently, the present study has incorporated the variable perceived enjoyment [4] in order to include constructs closely linked with the online game acceptance. Additionally, suspense keeps gamers in a heightened state of alert or flow experience, allowing to measure game rewards as perceived enjoyment [48]. In this manner, a relationship between perceived enjoyment and flow experience could be identified [49]. Thus, a higher perceived enjoyment will generate a greater flow experience [50]. Thus, the following hypothesis is presented:

**Hypothesis** **1** **(H1).** *Perceived enjoyment impacts positively on flow experience.*

In addition, perceived ease of use is associated with continuance intention [3,51,52,53,54]. Based on previous studies [4,10,14,15,16,21,55], the present study has proposed to adapt TAM, using the variable perceived enjoyment instead of perceived usefulness. According to researchers [4], perceived enjoyment is referred to the user perception about an ICT is inherently enjoyable. While perceived enjoyment is associated with intrinsic motivations, perceived usefulness is related to extrinsic motivations [14]. In hedonic ICT, like MMOG, perceived enjoyment is considered more powerful predictor for the continuance intention than the variable perceived usefulness [10,15]. At the same time, as it was said before, the construct perceived ease of use is related to the personal beliefs about the free of effort of an ICT [36]. In this respect, there is previous evidence that the ease of use of an ICT impacts positively on perceived enjoyment [16]. In this manner, the following hypotheses are defined:

**Hypothesis** **2** **(H2).** *Perceived enjoyment impacts positively on continuance intention.*

**Hypothesis** **3** **(H3).** *Perceived ease of use impacts positively on perceived enjoyment.*

Additionally, according to the TAM model, perceived ease of use is related to the continuance intention [3,14,20,52,53,54]. According to researchers [3], perceived ease of use in online games’ contexts could be considered as the user feeling about the easiness of their functions. It is a determinant of the continuance intention, as long as perceived ease of use is a good predictor of the behavioral intention to use [20,52]. In this line, there are many studies applied on several hedonic ICT fields that have demonstrated the connections between the constructs perceived ease of use and continuance intention [14,53,54]. Thus, the following hypothesis is drawn:

**Hypothesis** **4** **(H4).** *Perceived ease of use impacts positively on continuance intention.*

#### 2.2.2. Flow Experience

Flow experience is a state of concentration [18]. A person with this state ceases to perceive external stimuli, such as, temperature changes or noises [16]. This state is not exclusive to video gamers. Many athletes or artists achieve this status during the competition or the performance, when they omit the noise coming from the audience. The flow experience is divided into three constructs [19]: (1) focused attention, (2) control, and (3) curiosity. Online games can take great concentration by immersing the user in a virtual world [56]. In this respect, the flow experience in online games could measure the user attention during the game which would be related to the difficulty to play it [18]. Thus, flow experience could help to understand better the online game acceptance and its continuance intention to use. Thereby, flow experience state is only achieved when a person is completely concentrated on a specific task. It is usually assumed that these tasks are difficult and demand the whole user concentration [18]. In this manner, flow experience is related to continuance intention [4,15,19,56]. Supported on this, the following hypothesis has been proposed:

**Hypothesis** **5** **(H5).** *Flow experience impacts positively on continuance intention.*

#### 2.2.3. Needs for Autonomy, Competence, and Relatedness

SDT framework has been used widely in the gamification environment, insofar as it considers intrinsic and extrinsic motivations [57]. Concretely, the proposed model has incorporated three SDT needs: (1) competence, (2) autonomy, and (3) relatedness. First, competence is referred to the need of individuals to feel capable while successfully generating an outcome [21]. Besides this, autonomy involves the freedom of making decisions related to their own objectives [58]. In an online game context, gamers must make a wide range of decisions, even if each one is limited by a finite number of options. In this manner, many decisions in some games are predefined; however, they can satisfy the need for autonomy, appealing that they are associated with winning the game. In this manner, the need for autonomy is based in the freedom of choice [58]. 

In addition, the need for competence can be measured by self-efficacy. In this particular, according to researchers [52,59], perceived ease of use and training are related to self-efficacy. Based on that, the model combines the variables need for autonomy and competence, to the extent they measure the desire of achieving a goal, making their own decisions skillfully. For that, this variable would be related to perceived ease of use in the proposed model [37,46]. Therefore, the following hypothesis is proposed.

**Hypothesis** **6** **(H6).** *Needs for autonomy and competence impact positively on perceived ease of use.*

Furthermore, the need of relatedness is based on social interactions. At current, many online games have text and voice messaging services which allow the interactions between gamers. Indeed, many online games allow to form teams to achieve common goals. Thus, relatedness can be fulfilled even if the teammates are virtual non-player characters [58]. 

Based on the previous point, the need of relatedness is linked with the perceived enjoyment [21]. Moreover, the need for relatedness depends on social interactions. Thereby, the connection between social interactions and perceived enjoyment [16] is really associated to the need for relatedness [51,60]. In this manner, the following hypothesis is defined:

**Hypothesis** **7** **(H7).** *Need for relatedness impacts positively on perceived enjoyment.*

#### 2.2.4. Achievement and Challenge Emotions

Many investigations have used the TAM in multiple ICT environments, for instance, enterprise systems [52], smartphones [59], social media [53] or smart factory [61], among others. The present study has adapted the original TAM model to assess the online games acceptance, including the e-TAM variant. In this manner, flow experience, perceived enjoyment, perceived ease of use, and continuance intention has become the four core constructs of the proposed model.

e-TAM model [37] aims to include emotions that ICT can generate. The emotional variables incorporated in the e-TAM model are achievement and challenge. In fact, positive emotions lead gamers to the feelings of achievement and challenge. In this manner, it is relevant not only to identify, but also to measure the emotions that have been generated by the continuance usage of online games. At the same time, many online games characteristics incentive these positive emotions which motivate the use of them [12]. These characteristics may be, for example, obtaining badges or awards for achieving a specific objective [58]. 

Oppositely, negative emotions lead to the feelings of loss and deterrence [37]. Indeed, it could bring out negative feelings, such as, frustration, anxiety, or fear, which continuance intention of using the internet have very little significance in these negative emotions as demonstrated by a study [37], therefore not relevant for MMOG. In a similar way, the probability of winning the game increases suspense [48]. For that, the present study proposes to combine the achievement emotion and the challenge emotion variables. Both are related to the game enjoyment, considering that enjoyment is not based only on pleasure, but also achieving the completion of a task and suspense of winning as well [48]. Otherwise, continuance intention to use would allow to understand the attitude that a player has towards using the games again [37]. In this manner, achievement and challenge emotions are linked with the continuance intention to use.

**Hypothesis** **8** **(H8).** *Continuance intention impacts positively on achievement and challenge emotions.*

#### 2.2.5. Social Well-Being

There are many online game attributes, such as the avatar [58], through which an individual can, not only participate in virtual games, but also, they can create their own image, i.e., hair color, height, or weight, among other aspects. Within the virtual world of online games any disability that a user may have, with respect to the other gamers, is disregarded. For example, a disabled person can run a marathon in a video game just like a user who does not have a disability.

In this manner, the perceived value construct from the e-TAM model is based on the perceived utility [37]. However, the perceived value covers a greater number of technological elements, not only the benefits of performing a specific task. Indeed, it is relevant to measure the value that gamers place on online games, showing how important they are for themselves or for their communities. In the proposed model, perceived value will be included in the social well-being construct, as gamers use online games mainly for entertainment, relaxation, and fun with friends. Thereby, continuance intention is related to the social well-being [3,10,14,16,19,21,37,51,56]. Based on this model, the following hypothesis is drawn.

**Hypothesis** **9** **(H9).** *Continuance intention relates positively to social well-being.*

## 3. Methods

The present study has defined a PLS-SEM proposal to assess MMOG user acceptance. The application of the PLS-SEM method is recommended when the data have been obtained through surveys to assess the appropriateness of the dimensions of a model. According to researchers [62], SEM is a multivariate statistical method that allows to test a relational model with observable and unobservable dimensions. PLS-SEM can be used both for explanatory and predictive research [63]. 

PLS-SEM has some relevant differences with the covariance structural equation modeling (CB-SEM) technique. While both techniques try to test models with cause–effect relationships between latent constructs, they apply different procedures. In this particular aspect, PLS-SEM incorporates less restrictive requirements in the measurement of sample size scales and in the distribution of the data. At current, this method is widely accepted in all sciences.

In this manner, PLS-SEM does not need the conditions required by the traditional CB-SEM. In fact, the flexibility of PLS-SEM offers some advantages: (1) It can be used with small sample sizes, although a larger sample size increases precision, and (2) it is not necessary to assume a normal distribution of the data [64,65].

### 3.1. Participants

Participants were MMOG players. This study has target e-Sports gamers, such as Fortnite, League of Legends, Clash of Clans, Brawl Stars, among others. In particular, all of them were Fortnite players, but most of them played other eSports. Indeed, in the invitation letter, we requested that the responses were focused on Fortnite. When the potential participants agree to take part of the study, they received the URL with the questionnaire. The survey was conducted from February to June 2020. The participants were identified mainly using social networks, although some of them were also identified after visiting some internet cafés. A total of 268 questionnaires were obtained, from which these 264 responses were valid. This sample size exceeds the recommendations, considering a statistical power of 80% and assuming a 1% of significant level and a R^2^ = 0.1 [65].

Table 1 shows the demographic profile of the respondents. It is remarkable that more than 75% of users were 25 years old or younger and 85.17% were male. Moreover, more than 77% of gamers played at least once a day and 58.33% have spent money playing online games.

### 3.2. Instrument

A questionnaire was the instrument to collect the dataset. This instrument was designed based on relevant previous literature on the selected theoretical frameworks of the present study. Specifically, the measuring scale for the constructs included in the research model was defined with the measures from the sources shown in the Appendix A. Considering this measuring scale, an online questionnaire has been designed with Google Forms.

The first section of the questionnaire was used to obtain demographic information of the participants. In the second part, 27 items were incorporated in order to measure the eight constructs of the model: (1) autonomy and competence, (2) relatedness, (3) achievement and challenge, (4) flow experience, (5) perceived enjoyment, (6) social well-being, (7) perceived ease of use, and (8) continuance intention. Besides that, a five-point Likert-type scale was used for the participants to express their degree of agreement with the items on, being (1) “strongly disagree” and (5) “strongly agree”.

### 3.3. Data Analysis

The data analysis was structured into two steps: (1) SPSS v. 26 statistical software allowed to reach the descriptive statistics and the measurement reliability analysis, and (2) Smart PLS v. 3.3.2 software tested the PLS-SEM model. In addition, this software assessed through a factor analysis the correlation coefficient between dimensions.

## 4. Results

As a previous step to test the model, the validity of the measurement was carried out. Specifically, the reliability and the convergent and discriminant validity were verified. For that, an analysis of the Cronbach’s alpha and composite reliabilities was completed to evaluate the internal consistency reliability. Moreover, the convergent validity and discriminant validity for the latent constructs have been assessed with a confirmatory factor analysis (Table 2). In this manner, the reliability of measurement model is affirmed after observing that all item reliability results are above the suggested threshold value of 0.7. Then, the results for the convergent and discriminant validity are shown.

### 4.1. Convergent Validity

In this way, the item factor loadings were evaluated to measure the convergent validity. All of them were greater than 0.7, and all composite reliabilities for each construct in the model were well above 0.70. At the same time, the average variance extracted (AVE) exceeded 0.50 for each construct [66]. In a similar way, the loadings associated with reflective measures dimensions were higher than 0.70, meaning that more than half of the variance is captured by the constructs [67]. In this sense, the composite reliability measurements show a good internal consistency. In addition, the AVE values ranged from 0.653 to 0.851, well above 0.50. Therefore, all three conditions for convergent validity were met.

### 4.2. Discriminant Validity

Moreover, the requirements for assessing the discriminant validity are the following two: (1) the square root of the AVE must be higher than between correlations by dimensions [66] and (2) the factor loading of each indicator should be greater in the representative constructs than any other in the model [68]. On the one hand, the diagonal elements of the correlation matrix, show the square root of the AVE (Table 3), for a comparison purpose. Based on these values, the shared variances between two any dimensions of the model were lower than the variance of anyone. 

In addition, Table 4 shows the loading and cross-loading matrix. The values in bold show the highest loading for each item across all constructs. These results make evident that each item is a strong predictor to measure the intended construct, supporting, in this way, the discriminant validity of the scales in the proposed model. In this manner, the results allow to affirm the convergent and discriminant validity for all the constructs. 

### 4.3. Model Test

**Figure 2:**
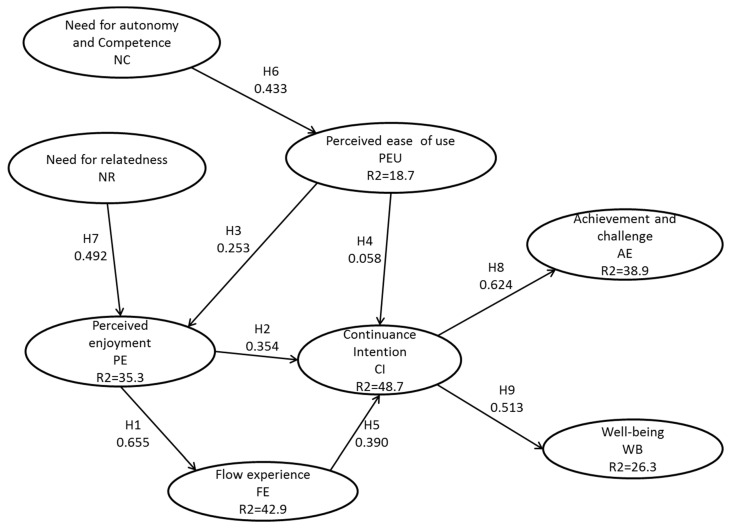
Path coefficient of the analysis.

The path coefficients of the model have been estimated with the Smart PLS software v. 3.3.2. The bootstrapping procedure with 5000 samples was used to generate the path coefficients relationship in the proposed model (Figure 2), and to calculate the variance explained. 

These results demonstrate that all the hypotheses were supported as the defined relationships are statistically significant [69], except for the H4. As shown in Figure 2, the proposed research model explains 48.7% of the variance of continuance intention to use online games, 26.3% of the variance in social well-being, 38.9% of the achievement and challenge, 42.9% of the variance in flow experience, 35.3% of the variance in perceived enjoyment, and 18.7% of the variance in perceived ease of use. 

In this sense, flow experience and perceived enjoyment were good predictors of continuance intention (β = 0.390, *p* < 0.001 and β = 0.354, *p* < 0.001, respectively), as continuance intention was for achievement and challenge emotions, and social well-being (β = 0.624, *p* < 0.001 and β = 0.513, *p* < 0.001, respectively). Additionally, need for autonomy and competence was a significant predictor of perceived ease of use (β = 0.433, *p* < 0.001). In addition, perceived ease of use and need for relatedness have impacted on perceived enjoyment (β = 0.253, *p* < 0.001 and β = 0.492, *p* < 0.001, respectively). Furthermore, this study identified perceived enjoyment as a significant predictor for flow experience (β = 0.655, *p* < 0.001). However, perceived ease of use is not a significant predictor of continuance intention (β = 0.058, *p* > 0.05). Table 5 summarizes the hypotheses’ test. 

## 5. Discussions

This study has focused on two objectives: (1) proposing an acceptance model to predict the continuance usage of MMOG, and (2) knowing how this continuance usage encourages social well-being. In this manner, a PLS-SEM model has been defined using a combination of the TAM, its variant e-TAM and SDT, comprising eight constructs: (1) autonomy and competence, (2) relatedness, (3) achievement and challenge, (4) flow experience, (5) perceived enjoyment, (6) social well-being, (7) perceived ease of use, and (8) continuance intention. In this respect, scarce evidence has been found to reach responses to both objectives, which, in short, intend to analyze the motivations of MMOG use. Thereby, the present study has helped to contribute to the topic about the acceptance of this type of online game.

In this manner, the findings have covered the first of the objectives. In particular, the results have revealed a measurement and structural model to predict continuance intention to use MMOG. In addition, this model has incorporated some factors that affect behavioral intention to use MMOG. Indeed, the results have supported the represented hypotheses in the research model, except for the hypothesis H4. In this way, these results are in line with previous research. Based on prior investigations, such as studies [21] and [50], the present model has incorporated perceived enjoyment and flow experience as the main constructs related to the continuance intention of online games, especially MMOG. In fact, the 48.7% of the continuance intention is explained by the research model. 

As it was already described by some investigations [10,15], perceived enjoyment is confirmed as a powerful predictor for the continuance intention of MMOG. In a similar way, it has been demonstrated that flow experience in online games explain the continuance intention due to this concentration state which draw users’ attention during the game. This fact makes MMOG users motivated to continue using it. In addition, these results are similar, or even better, to the findings of other studies in the same field. Thus, other proposed models of previous studies have explained the variance of the continuance intention to play online games in 50% [11], 47% [13], and 34.2% [22]. In this particular aspect, these results indicate that online games are considered entertaining, as it has already been established by research, such as studies [4,10,14,15,16,21,55], as long as they are ease to use [10] and possess a clear set of rules [18]. 

Furthermore, the findings have shown that the flow experience state can only be achieved when the abilities of the gamer match necessary skills on online game. Likewise, when gamers achieve the flow experience state, it gives a feeling of having done something right [18], which drives them to continue playing. In this sense, perceived enjoyment has been able to explain the 42.9% of flow experience. This result is higher than a previous study [11], in which enjoyment manages to explain the 21% of flow experience. At the same time, the 35.3% of the perceived enjoyment has been explained by the model. Besides that, these results indicate that MMOG that are easy to use do not have to generate the desire to continue using them, as the perceived ease of use does not bring satisfaction by itself. This suggestion was already indicated previously [70], but unlike in the present study, it was referred to mobile augmented reality apps. In this sense, the present results indicate, in line with a previous study [71], that it should be considered that a utilitarian factor does not encourage the use of online games as hedonist factors do.

However, flow experience explains continuance intention in a more significant way that the other factors [56], supporting the hypothesis H5, which was based on studies, such as [4,15,19], with a path coefficient of 0.390, greater than studies like [11]. In this respect, flow experience does not only drive the gamer to continue playing online games, but it also increases the satisfaction in the whole experience as they mentally transport themselves to a virtual world, merging action and the self-awareness loss [18]. Thus, enjoyment encourages the flow experience (hypothesis H1), as it was suggested in prior literature [49,50], with a path coefficient of 0.655, highest than other studies [11]. 

In addition, as it has been evidenced on previous literature, competence and autonomy are not related to perceived usefulness or perceived enjoyment, but to the perceived ease of use [37,46]. Therefore, the present study has supported the hypothesis H6, as those that defined the relationships between the need for autonomy and competence with perceived ease of use. However, the need for autonomy and competence only explains 18.7% of variance for the perceived ease of use. This could be explained as there are probably other factors that influence this construct, like experience, knowledge, among others.

In a similar way, the need for relatedness has had a significant impact on perceived enjoyment [21], supporting the hypothesis H7, with a path coefficient of 0.492, greater than previous studies, such as [17] and [72]. In this way, it can be highlighted the communication capabilities of online games, because of they are designed under a collaborative model. Besides this, it can be suggested that a good attitude towards continuance intention will encourage satisfaction [73]. Nonetheless, continuance intention drives positive emotions such as challenge, achievement, and social well-being, and not negative ones. This idea was supported in prior studies [37], and it was defined in the hypotheses H8 and H9. In this respect, continuance intention explains the 26.3% of social well-being variance, which is greater than other similar studies [17]. In this respect, it is important to highlight that some studies have defined hypotheses to test the impact of achievement emotions on the continuance intention, but it was not supported [11] or had a very low impact [22]. The present study has tested the inverse relationship and it was supported, showing a relevant contribution. In addition, continuance intention has had the ability to explain the 38.8% of the variance of the achievement and challenge construct. These results suggest that to continue playing MMOG bring positives emotions and a better social well-being rather than negative ones. In fact, even when gamers lose, they experience the feeling of challenge, to do it better the next time. These results answer the second aims, which sought to know how the continuance usage encourages social well-being.

Definitely, the main purpose of this study has been to understand the MMOG acceptance. In this manner, this study provides an explanation to predict the continuance intention, offering the academic community and companies about the emerging trends of online games. In this manner, one of the main contributions of the present study has been to combine the e-TAM approach developed by researchers [37] in 2019 with SDT. In fact, this proposal has arisen significant results since the model broadly explains the proposed constructs. In this way, the present study has allowed to verify the proposed theoretical frameworks that offer an adequate context to explain the use of ICT for entertainment, as in the case of online games, as well as the emotions associated with this type of ICT.

## 6. Conclusions

The present investigation has proposed that the spreading of MMOG use is determined in part by motivations, which support the activation of a positive attitude towards continuing use of MMOG. In particular, the incorporation of constructs in the research model, such as perceived enjoyment and flow experience, has been key to improve the knowledge regarding the gamer acceptance. Moreover, the construct need for autonomy and competence has supported the identification of intrinsic motivations that have contributed to explain continue intention to play MMOG, and how it encourages social well-being. 

This research has demonstrated that extrinsic and intrinsic motivations play a relevant role on gamers´ continuance intention to play MMOG. In this way, the present study has been able to accomplish its objectives, developing a model able to understand and predict the continued use of online games, in addition to identifying how this continuance usage boosts social well-being. Bearing this in mind, some contributions and limitations can be extracted.

On the one hand, as the first contribution, this article has provided some empirical evidence for the development of investigations which propose models based on motivations to assess the usage intention of online games. Moreover, the described findings and discussions could be useful for the online gaming industry. In this respect, the results may be a support for defining those game functionalities that allow to retain gamers and improve the flow experience. In this sense, these results suggest to the gaming industry to consider the incorporation of the studied motivations in the online game design, in order to stimulate and incentive its use. On the other hand, as the second contribution, the present study can help to understand the online gamer psychology, and thus be able to raise research questions related to its mental health.

These contributions allow to define two recommendations for practitioners. On the one hand, gamers should select those MMOG that reinforce their psychological needs and well-being in order to improve the personal development. In this manner, MMOG should be an excellent driver to enhance skills and to correct psychological disorders. On the other hand, MMOG could become a way to encourage social interactions, covering the need to feel part of a social group.

Finally, some limitations are identified. First, the study could have used a larger sample to increase the robustness of the results. Second, the relations between the perceived ease of use with perceived enjoyment have had a low significance, suggesting that there are other constructs that have not been included in the study. In this manner, future studies could research additional aspects of virtual communities and online games that could influence on the social well-being construct, Moreover, these results show that the competitive aspects of e-Sports, as well as the extrinsic motivation professional gamers have had a reduced impact on the literature, and they require a greater number of investigations to improve their knowledge.

## Figures and Tables

**Table 1:** Profile of participants.

Dimensions	Options	Number of Responses
Gender	Male	225 (85.2%)
	Female	39 (14.77%)
Age (years old)	<20	104 (39.4%)
	≥20 and ≤25	98 (37.12%)
	≥26 and ≤30	27 (10.27%)
	≥31 and ≤35	7 (2.65%)
	≥36 and ≤40	11 (4.16%)
	>40	17 (6.43%)
Online Games use frequency	Rarely	20 (7.58%)
	Once a week	39 (14.77%)
	Once a day	114 (43.18%)
	Several times a day	91 (34.47%)
Spent money	No	110 (41.67%)
	Yes	154 (58.33%)
Type of MMOG	Fortnite	264 (100%)
	League of Legends	125 (47.3%)
	Clash of Clans	86 (32.5%)
	Brawl Stars	93 (35.2%)
	Minecraft	56 (21.21%)
	Dota 2	146 (55.3%)
	Roblox	75 (28.4%)
	Counter Strike	43 (16.2%)

**Table 2:** Item loadings and measurement reliability.

Latent Constructs and Items	Item Reliability	Composite Reliability	Cronbach’s Alpha	Average Variance Extracted (AVE)
Achievement and challenge (AE)		0.882	0.823	0.653
AE1	0.874			
AE2	0.735			
AE3	0.844			
AE4	0.772			
Continuance Intention (CI)		0.920	0.825	0.851
CI1	0.920			
CI2	0.925			
Flow experience (FE)		0.859	0.754	0.670
FE1	0.857			
FE2	0.827			
FE3	0.769			
Need for autonomy and competence (NC)		0.925	0.898	0.711
NA1	0.844			
NA2	0.843			
NC1	0.782			
NC2	0.855			
NC3	0.889			
Need for relatedness (NR)		0.906	0.844	0.763
NR1	0.898			
NR2	0.885			
NR3	0.837			
Perceived enjoyment (PE)		0.889	0.834	0.667
PE1	0.771			
PE2	0.840			
PE3	0.843			
PE4	0.811			
Perceived ease of use (PEU)		0.881	0.798	0.713
PEU1	0.846			
PEU3	0.879			
PEU4	0.806			
Social well-being (WB)		0.903	0.841	0.757
WB3	0.871			
WB1	0.871			
WB2	0.868			

**Table 3:** Measurement model estimation.

Construct	AE	CI	FE	NC	NR	PE	PEU	WB
AE	**0.808**							
CI	0.624	**0.923**						
FE	0.677	0.636	**0.819**					
NC	0.590	0.532	0.584	**0.843**				
NR	0.537	0.471	0.536	0.571	**0.874**			
PE	0.712	0.630	0.655	0.519	0.540	**0.817**		
PEU	0.355	0.278	0.250	0.433	0.190	0.346	**0.844**	
WB	0.618	0.513	0.575	0.583	0.671	0.529	0.137	**0.870**

Note: Diagonal components are the square root of the AVE.

**Table 4:** Loading and cross-loading matrix.

Item	AE	CI	FE	NC	NR	PE	PEU	WB
AE1	**0.874**	0.524	0.608	0.506	0.476	0.627	0.308	0.551
AE2	**0.735**	0.379	0.471	0.292	0.292	0.469	0.161	0.400
AE3	**0.844**	0.600	0.476	0.540	0.486	0.643	0.408	0.511
AE4	**0.772**	0.476	0.647	0.525	0.449	0.535	0.221	0.521
CI1	0.556	**0.920**	0.604	0.486	0.397	0.631	0.251	0.418
CI2	0.595	**0.925**	0.571	0.496	0.471	0.533	0.263	0.527
FE1	0.585	0.635	**0.857**	0.483	0.399	0.575	0.232	0.421
FE2	0.591	0.445	**0.827**	0.556	0.514	0.488	0.201	0.553
FE3	0.487	0.458	**0.769**	0.404	0.419	0.537	0.175	0.458
NA1	0.513	0.457	0.513	**0.844**	0.491	0.457	0.354	0.527
NA2	0.518	0.461	0.511	**0.843**	0.473	0.421	0.413	0.527
NC1	0.464	0.420	0.482	**0.782**	0.422	0.442	0.388	0.390
NC2	0.514	0.445	0.488	**0.855**	0.495	0.447	0.315	0.496
NC3	0.472	0.456	0.457	**0.889**	0.530	0.418	0.332	0.515
NR1	0.502	0.458	0.508	0.512	**0.898**	0.474	0.183	0.529
NR2	0.427	0.334	0.419	0.512	**0.885**	0.450	0.184	0.558
NR3	0.475	0.436	0.472	0.473	**0.837**	0.488	0.132	0.666
PE1	0.526	0.515	0.435	0.426	0.420	**0.771**	0.275	0.353
PE2	0.549	0.462	0.499	0.391	0.412	**0.840**	0.207	0.477
PE3	0.634	0.525	0.604	0.419	0.498	**0.843**	0.250	0.491
PE4	0.606	0.547	0.582	0.455	0.427	**0.811**	0.388	0.402
PEU1	0.318	0.269	0.204	0.342	0.184	0.323	**0.846**	0.164
PEU3	0.293	0.196	0.185	0.332	0.178	0.268	**0.879**	0.120
PEU4	0.286	0.233	0.238	0.415	0.120	0.281	**0.806**	0.065
WB3	0.567	0.514	0.494	0.483	0.536	0.493	0.152	**0.871**
WB1	0.506	0.418	0.506	0.515	0.611	0.440	0.079	**0.871**
WB2	0.532	0.387	0.503	0.531	0.618	0.438	0.120	**0.868**

**Table 5:** Test of the hypotheses.

	Path Coefficient	t-Value	Supported
H1: PE -> FE	0.655	16.338 ***	Yes
H2: PE -> CI	0.354	4.870 ***	Yes
H3: PEU -> PE	0.253	4.141 ***	Yes
H4: PEU -> CI	0.058	1.181	No
H5: FE -> CI	0.390	5.403 ***	Yes
H6: NC -> PEU	0.433	6.713 ***	Yes
H7: NR -> PE	0.492	10.016 ***	Yes
H8: CI -> AE	0.624	13.396 ***	Yes
H9: CI -> WB	0.513	10.717 ***	Yes

Significant at: *** *p* < 0.001; t(0.001;∞) = 3.3195.

## Data Availability

The data presented in this study are available on request from the corresponding author.

## Appendix A. Questionnaire

**Table A1:** Questionnaire.

Construct	Items	Description	Source
Achievement and challenge	AE1	Using the online game gives me satisfaction.	[74]
	AE2	Using the online game gives me pleasure.	
	AE3	Using the online game gives me enjoyment.	
	AE4	By using online games, I have the experience that everything flows.	
Continuance intention	CI1	I intend to continue using the online game in the future.	[75]
	CI2	I plan to continue using the online game frequently.	
Flow experience	FE1	By using the online games, I keep my attention completely focused on it.	[76]
	FE2	Using the online game makes me feel in control.	
	FE3	I am curious to use the online game.	
Need for autonomy and competence	NC1	Using the online game increases my competence.	[77]
	NC2	Using the online game improves my feeling of being capable to do things.	
	NC3	Using the online game improves my feeling of being capable to reach complex aims.	
	NA1	Using the online game allows me to do activities in an alternative way.	
	NA2	Using the online game allows me to do activities as I desire.	
Need for relatedness	NR1	Using the online game integrates me in a group.	[77]
	NR2	Using the online game allows me to make new relationships with people.	
	NR3	Using the online game allows me to contact with people to converse about things that interest me.	
Perceived enjoyment	PE1	Using the online game is enjoyable.	[78]
	PE2	I have fun while using the online game.	
	PE3	I experienced excitement while using the online game.	
	PE4	The actual process of using the online game is pleasant..	
Perceived ease of use	PEU1	Using the online game is clear and easy to understand.	[75]
	PEU3	I find the online game to easy to use.	
	PEU4	I find the online game easy to get what I want it to do.	
Well-being	WB1	The online game is important for my social well-being.	[79,80]
	WB2	The online game is important for the improvement of the quality of my life in my community.	
	WB3	Overall, what is the importance of online games in your life?	
